# A long-term negative effect of monetary incentives on the participatory surveillance of animal disease: a pilot study in Chiang Mai, Thailand

**DOI:** 10.1186/s12889-022-14837-8

**Published:** 2022-12-29

**Authors:** Tossapond Kewprasopsak, Charuk Singhapreecha, Terdsak Yano, Reiner Doluschitz

**Affiliations:** 1 Department of Farm Management, Division of Computer Applications and Business Management in Agriculture (410 c), University of Hohenheim, Stuttgart, Germany; 2 Faculty of Economics, Chiang Mai University, Chiang Mai, Thailand; 3 Center of Excellence in Econometrics, Faculty of Economics, Chiang Mai University, Chiang Mai, Thailand; 4 Faculty of Veterinary Medicine, Chiang Mai University, Chiang Mai, Thailand; 5 Integrative Research Center for Veterinary Preventive Medicine, Faculty of Veterinay Medicine, Chiang Mai University, Chiang Mai, Thailand

**Keywords:** Animal health, Behavioral economics, Incentive, Monetary market, Participatory disease, Surveillance, Social market

## Abstract

**Background:**

In general, animal diseases have a significant impact on public health; accordingly, an effective animal disease surveillance system is an important control system that requires efficient and engaging participants in the long run. The purpose of this study is to assess the impact of monetary and social motivation on animal disease surveillance. We hypothesized that there are two sorts of motivation based on Fiske's relational theory (1992): monetary incentives (monetary markets) and nonmonetary incentives (social markets).

**Methods:**

In Chiang Mai Province, Northern Thailand, we analyzed data from a pilot project that began in 2014 and used a mobile application to report on signs that identify animal health problems. A total of 67 participants from 17 different areas in the central part of the province participated in this study. Participants in this study were divided into two groups: those who received monetary incentives and those who received social incentives.

**Results:**

According to the findings, the monetary market group's effort was significantly higher than that of the social market group during the time when the volunteers in the monetary market group were paid. However, in the long run, the monetary market group reported significantly less than the social market group. Social incentive, on the other hand, was more efficient once the payment period ended.

**Conclusions:**

Social incentive outperformed monetary motivation in terms of efficiency and sustainability in the long run. Not only did the volunteers who were offered monetary incentive put in less effort than those who were offered the social incentive, but they were also not remotivated by the social incentive after the payment period had ended.

## Background

The incidence of animal diseases in the agricultural sector can increase costs and reduce profitability significantly. It can contribute to an increase in the vulnerability of farming. Hence, the disease surveillance system, as a practice, is an important part of diseases control. Such a system includes reporting diseases or signs and symptoms and analyzing the data. Disease reporting is the first stage of the disease surveillance system, and its subsequently induces other processes. It can lead to major early detection and rapid response activity. Therefore, the World Organization for Animal Health (OIE) formally indicated that OIE country members have a duty to report their country’s animal disease situation to guarantee transparency and to improve knowledge of world animal health [1]. At a local level, farmers and livestock owners play an important role in reporting animal diseases. Compared to veterinarians, they come into closer contact with animal health. They can provide to authorities important data that indicate the population and the area at risk. Such data can then be used in the animal disease surveillance system. There are several types of disease surveillance, including passive and active surveillance [2]. The Passive surveillance is the most common system, which have been used in animal health surveillance, because it is inexpensive approach. The veterinary authorities select only the relevant data for application in the animal disease surveillance system from their routine work and the reports from farmers or other sources. For this reason, passive surveillance is inexpensive and commonly used by the veterinary authorities. On the other hand, active surveillance needs to be designed and conducted by veterinary authorities with the specific objective of the disease surveillance system. Such a system involves spending a great deal of time, labor, and budgetary resources [3, 4].

The participatory disease surveillance system is a form of active disease surveillance system in which public health agencies collaborate with involved partners and stakeholders to produce health and disease data. The participatory disease surveillance system is less cost prohibitive and more adaptable than other forms of active surveillance systems. [3]. Furthermore, it can also be applied on a broad scale, enabling low-cost animal population-based monitoring. Community members who are willing to respond quickly by providing health data and who can contribute informative, insightful data about health behavior are crucial stakeholders in the participatory disease surveillance system [5]. Furthermore, modern technology, especially smartphones, empowers individuals and communities to communicate data more quickly [6].

The Participatory One Health Disease Detection (PODD) was a pilot initiative that included digital technologies, local government agencies, and community volunteers. The project assembled a team of 296 volunteers from 74 different local government entities [7]. Through the use of smartphones, these volunteers contributed significantly to the surveillance system developed by the PODD initiative to record unusual animal illnesses and fatalities, livestock diseases, animal attacks, food safety issues, human diseases, and environmental issues.

The performance and effectiveness of the participatory disease surveillance system are dependent on motivation of participants. This study includes a variety of motivational explanations. Physiological needs, safety needs, belongingness and love needs, esteem needs, and self-actualization needs were identified by Maslow in 1943 as the five levels that drive individuals to attain their targets [8]. It is indisputable, however, that today's use of money as a motivator is prevalent. The reason for this is because we live in an age of market triumphalism, in which money can purchase most of our desired items [9]. Human beings as laborers, for example, will provide their time and effort in exchange for wage under the standard economic model of labor [10].

Employees work more for high incomes and less for lower incomes, according to the classic labor model of economics. In other terms, there is no labor if there is no payoff. Therefore, the overriding issue is, what ought we to do? Is money the only successful way to motivate partners in a participative system, or is there a non-monetary motivating strategy that is even more efficient and durable? Although a participative disease surveillance system has also been implemented, the current disease epidemic circumstances have a significant influence. Throughout 2005, for instance, zoonoses such as avian influenza afflicted populations in Southeast Asia. According to modeling study projections, the next pandemic might infect and kill between 2 and 7.4 million people globally [11]. Economic losses, human and animal health issues, and food insecurity are all possible outcomes of these projections. The future of such pandemics has remained unclear since human vaccinations, such as influenza or avian influenza vaccines, are not yet available for broad use because prototype vaccines are currently being developed [12]. As a result, we need not just improved effective vaccine and vaccinations, but also a speedy alarm system for early diagnosis so that problems may be solved by reducing transmission of pathogens before a pandemic occurs. The monitoring system, on the other hand, is a continuous activity that must be sustained. As a consequence, the PODD project faced the challenge of motivating volunteers, especially in the long run.

We sought to explain the short-term and long-term behaviors of participants using the concepts of monetary and nonmonetary incentives in this study. The theoretical framework of this study is composed of monetary and nonmonetary incentives, which are referred to as monetary markets and social markets, respectively [13]. Fiske's relational model theory (1992) was used to develop the notion of monetary and nonmonetary incentives. Fiske studied various types of human relationships, by searching studies from a variety of societies. Human relationships are divided into four categories: community sharing (CS), authority ranking (AR), equality matching (EM), and market pricing (MP). These four categories of relationships encompass not just society relationships, but also relationships within a social group or between individuals [14]. Fiske's relational models were rearranged in 2004 by Heyman and Ariely, which combined the CS, AR, and EM. Social markets [13] was the name given to this combination. Only MP was referred to as monetary markets.

People in social markets have a well-balanced human interaction and prioritize altruism. People offer their time and energy without expectation of reciprocation. If there would be a reciprocation at some activities, it is not always instantaneous, nor does it have to be equivalent to the time or effort invested. Volunteer caregivers, for example, offer to elderly care without expectation of reciprocation. They usually receive nothing, except appreciation in return. People are more clear and direct in monetary markets because they calculate the costs and benefits of every action, including opportunity costs, which are the expenses of losing another alternative once one is chosen. The return must be direct and obvious, with no ambiguity, and it should cover or surpass the cost. If an elderly person requires assistance, for example, hourly payments must be provided. The providing care finally ends when the compensation is discontinued.

Previous studies demonstrated that when the social and monetary incentives must interact with each other, monetary incentives are more influential. this means that social incentives will disappear for a long time, and it is difficult to return to social incentives after money incentives are used [10, 15, 16]. Therefore, one must carefully consider the consequences of using monetary incentives, especially in the case of social activities that require volunteer groups. Monetary incentives make volunteers compare their compensation to the normal wage rate and their expectations which are equal to the amount of compensation [17]. Moreover, monetary incentives can reduce effort after compensation is terminated [18, 19]. In addition, monetary incentives make it easy to push people because such incentives make people think about money even though there are no real payments [20]. Therefore, compensation payments are enough to shift volunteers from social incentives to monetary incentives.

At the beginning of the PODD project, monetary incentives were used to stimulate the surveillance system in the short run. If the system was functional, it would be expanded to other provinces in Thailand. However, the project could not provide the monetary incentives in the long run or in the other provinces. The pilot project and interested local governments could use the results of this experiment to design incentive policies for the surveillance reporters in the other areas to which system is expanded. As the results of reviewing the literature, the main objective of this study is to compare and describe the effect of monetary and social incentives on participants’ efforts during the course of the study. The specific objectives could be identified as follows:

- Objective 1: To compare and describe the effects of monetary and social incentives during the period when compensation was paid.
- Objective 2: To compare and describe the effects of monetary and social incentives at the end of the payment period.
- Objective 3: To compare and describe the effects of monetary and social incentives in the long term after compensation was terminated.

## Methods

### Study design

**Fig. 1:**
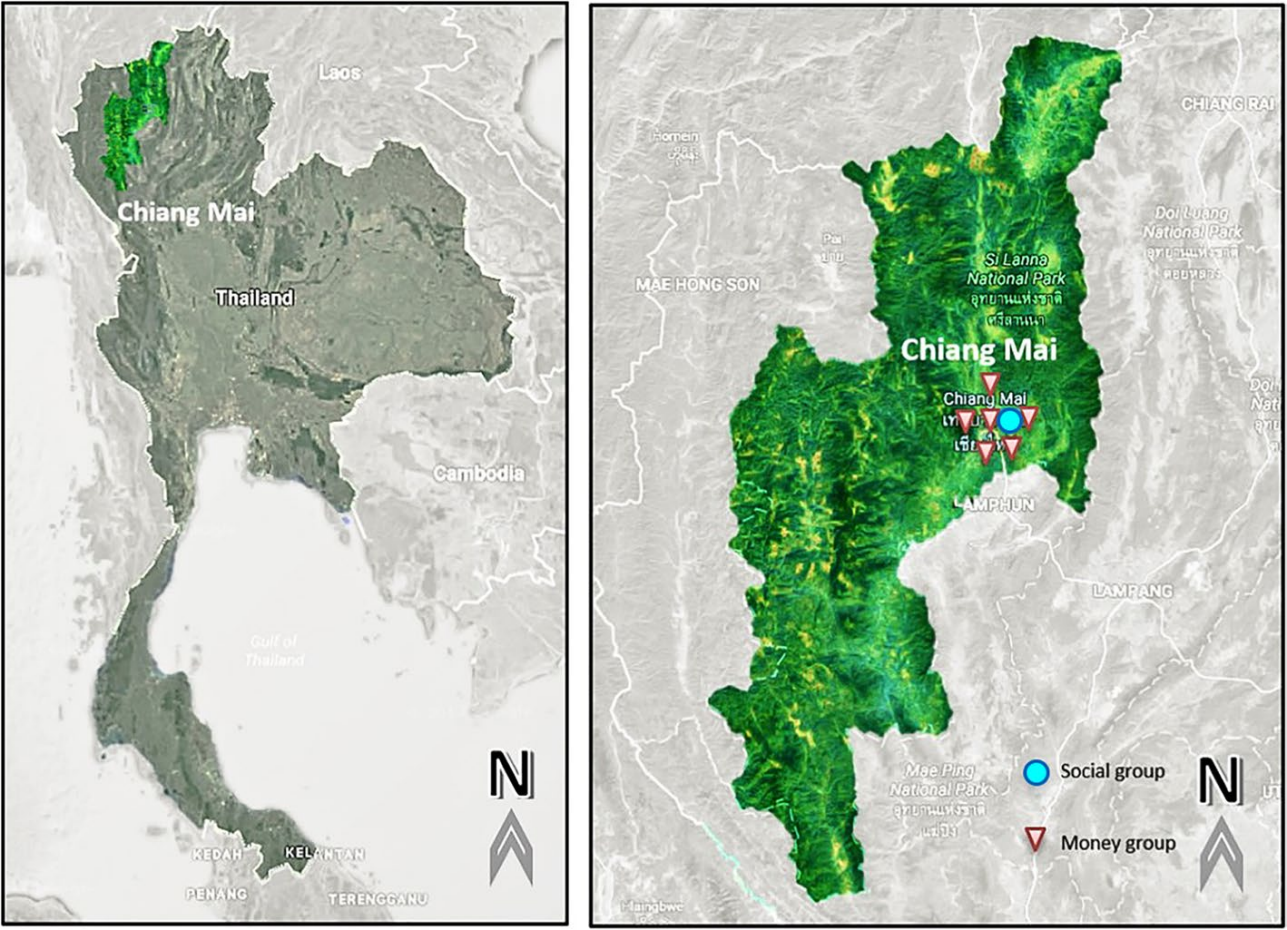
Study Area and Sample area in the Chiang Mai Province of Northern Thailand. Source: Own figure after Google map

This study used data from the PODD project, which was carried out in the Chiang Mai province, where the pilot was carried out. Backyard livestock, especially chicken and cattle, were produced in the study area. In residential areas, chickens are allowed to roam freely. They are allowed to roam about looking for food such as worms, insects, or rice, and are given rice at the end of the day by their owners. Chicken and cattle are maintained for the purpose of money saving reason and then sold when the owners require the income. The participants in this study are 296 PODD volunteers who have been involved in the project from its inception in 2015. All the experiment protocol for involving human data in this study was in accordance to the Declaration of Helsinki. The geographical structure of the province, which is generally mountainous, is the study's limiting constraint (Fig. 1). This constraint has an impact on the signal quality of the internet. Furthermore, different parts of the province had various livestock and population densities. Due to our limited constraint, we were unable to conduct systematic sampling of all of the participants in the PODD study. We selected 67 volunteers from 17 different study areas for this study (3 to 5 volunteers per area). To minimize the effect of other conditions, such as the diversity of livestock, population densities, and the internet connection, which is our limiting condition, all volunteers located in the central part of Chiang Mai Province.

### Procedures

The group selection was not random because of the limitation of the pilot study. In the beginning period of the PODD project, all volunteers in the project received either monetary incentives or social incentives. The monetary incentives were monthly payments throughout the duration of the pilot project (2014–2015), monetary support for internet and call charges monetary support, and a smartphone. The social incentives were being informed of the benefits of their report and surveillance system for their community and society (public good). Moreover, the project arranged a certificate as a reward for the volunteers who regularly and consistently reported.

. In the center of the urban area of Chiang Mai Province, there were 18 volunteers, who rejected receiving all kinds of monetary incentives. This means that they participated in the project based on only social incentives. They wanted to participate in the project out of a social duty to improve the quality of public health and animal health in their community. These groups were used as the control groups in this study.

Participants in this study were separated into two groups (Table 1). The experimental group consisted of 49 volunteers from 12 different areas who received a monthly payment of 400 baht ($11.5). The cost for a smartphone ($54.5) was paid by the project, as well as compensation for monthly internet and mobile rates. Regardless of whether they reported or not, this group of volunteers received paid monthly until the payment period ended. The monetary incentive brought this group of volunteers to the experiment, and they were notified of their monthly wage and other benefits. The control group consisted of 18 volunteers from five different areas who were not compensated (no monthly payment, a smartphone, or compensation for monthly internet and mobile rates). This group of volunteers was engaged in the study with no monetary incentive. Social incentives were used to entice them to engage in this study, and they used their own smartphones and internet subscriptions to install the PODD app and report.

**Table 1:** The number of reports per month according to group of volunteers

Group of volunteers	Number of volunteers	Reports per month			
		Period 1^a^	Period 2^b^	Period 3^c^	Period 4^d^
Experimental group: (Monetary Group)					
1	5	17	14	0	9
2	4	19	13	17	2
3	4	21	23	9	9
4	4	23	13	16	5
5	4	25	20	15	6
6	4	22	10	5	6
7	4	24	26	25	19
8	4	28	25	24	8
9	4	30	29	27	6
10	4	24	17	0	0
11	4	31	28	23	0
12	4	27	31	24	8
Control group: (Social Group)					
1	4	12	21	18	10
2	4	30	12	11	8
3	4	13	26	26	17
4	3	11	19	9	18
5	3	20	20	10	18

Sources: Computation and analysis from PODD volunteers report database

^a^Period 1 refer to the period of 5 months before the end of compensation

^b^Period 2 refer to the period of 1 month after the end of compensation

^c^Period 3 refer to the period of 5 months after the end of compensation

^d^Period 4 refer to the period of 10 months after the end of compensation

At the beginning, the project decided to use only monetary incentives for all of the volunteers who reported in the project. This means that in the monetary market group, the volunteers had been motivated by monetary activities since the beginning of their participation in the project. There are no data from before compensation was started. For this reason, this study had to use the social market group data to compare the impact of monetary and social incentives.

Both groups have been briefed about the advantages of the PODD project to public and were educated by the project with the similar information about animal outbreaks, the importance of reporting, and how to install and submit their reports through smartphone apps. It was their responsibility to report both normal and extraordinary animal-related happenings in the communities they were in charge of. Taking pictures, sharing the location, and observing using the smartphone app were all included in their reports. This experiment collected data on animal health abnormalities from four types of livestock: backyard chickens, pigs, dairy cattle, and beef cattle. The study taught the participants how to detect the fundamental signs of significant outbreak as Newcastle disease in poultry, porcine reproductive and respiratory syndrome (PRRS) in pigs, and foot-and-mouth disease (FMD) in cattle. In normal conditions, they would have to report a regular occurrence to ensure that there were no abnormal animal-related incidents in the community. Whether or not they had observed any signs of animal illnesses, they were required to report daily [7].

The only project intervention that differed between the groups were the compensation in the form of monthly salary payments, and the provision of a smartphone with free calls and internet for the period of the study. We designed this study to answer the following three study questions based on our objectives:

- Question 1 During the period when compensation was paid, were the efforts of the volunteers in the monetary market group higher than those of the volunteers in the social market group?
- Question 2: Did the efforts of the volunteers in the monetary market group decrease at the end of the payment period, and in contrast, did those of the volunteers in the social market group not decrease over the same period of time?
- Question 3: Did the volunteers in the monetary market group perform less well than those in the social market group in the long term after the compensation had ceased?

The first question examines the impact of monetary incentives (compensation and a smartphone with free of charge) on volunteers in the group of monetary incentive. We assume that the monetary market group makes better effort than the social market group. The second question concerns comparing the efforts of both groups over a lengthy amount of time. Compensation has ceased to be paid. The second question concerns comparing the efforts of both groups over a long term. In this question, we assume that the social group's effort remained constant throughout time, but the monetary market group's effort declined once compensation has ended. Finally, the third question concerns the long-term impact of monetary incentives. In this case, we assume that the monetary incentive has a negative long-term effect and decreases the efforts of the monetary market group. Furthermore, after the monetary incentive was eliminated, this question examines whether volunteers continued their behaviors based on the monetary market or returned to the social market.

### Instruments

The PODD automated system, which was linked to the Department of Livestock and the local government, collected the data submitted by both groups on the PODD application. Every volunteer in both groups was given their own ID to use when logging into the PODD application. Their monthly reports were measured based on the number of reports they submitted for each of the areas for which they were responsible. (Table 1). Furthermore, because our study focused on the rate of incidences, we calculated average monthly reports by counting just whether or not a report was submitted, implying that incidence variation had no impact on the average monthly reports. The highest number of reports each month was 30–31, while the minimum was zero. The project's accessible data covered a two-year period from 2015 to 2016. Because it took a lot of time to build up the system for each collaborating local government, such as teaching volunteers using the PODD application and supporting them with fundamental understanding of animal diseases in their local area (prepared by PODD vets), establishing local officials' computers to monitor reported data, and teaching the authority how to utilize and interpret the dashboard map's surveillance system, in a different period of time, the PODD project was used as a surveillance system in each area of Chiang Mai Province. The central part of Chiang Mai was chosen to minimize the effects of mobile signal variance, particularly in upland areas, as well as the effect of density and diversity of livestock species in other parts of the province. This area was added to the project near the ending of the compensation period. After offering training in all the monitoring system's operations, this area was fully functional five months before the ending of the compensation period. As a result, our study chose to start comparing data from this time period.

### Statistical analysis

To analyze the data of this study, the outcome variable in this study is the number of reports per month. The number of reports per month was reported as the mean, and repeated-measurement ANOVA was used to test the significant interaction of treatment and time. The *p* value was set to 0.05. The number of reports per month from each group was separated into four periods of time: Period 1 (five months before the end of compensation), Period 2 (one month after the end of compensation), Period 3 (five months after the end of compensation), and Period 4 (10 months after the end of compensation). The values at each single point of time were compared by a t test (paired analyses). To demonstrate significant differences, the level of statistical significance was set to a *p value* of 0.05.

## Results

To obtain the results of this study, we tested the normality, the correlated error, and homogeneity using the data from the four periods of time, the duration of each of which was a five-month interval. These data did not violate the assumption before conducting repeated-measurement ANOVA.

**Fig. 2:**
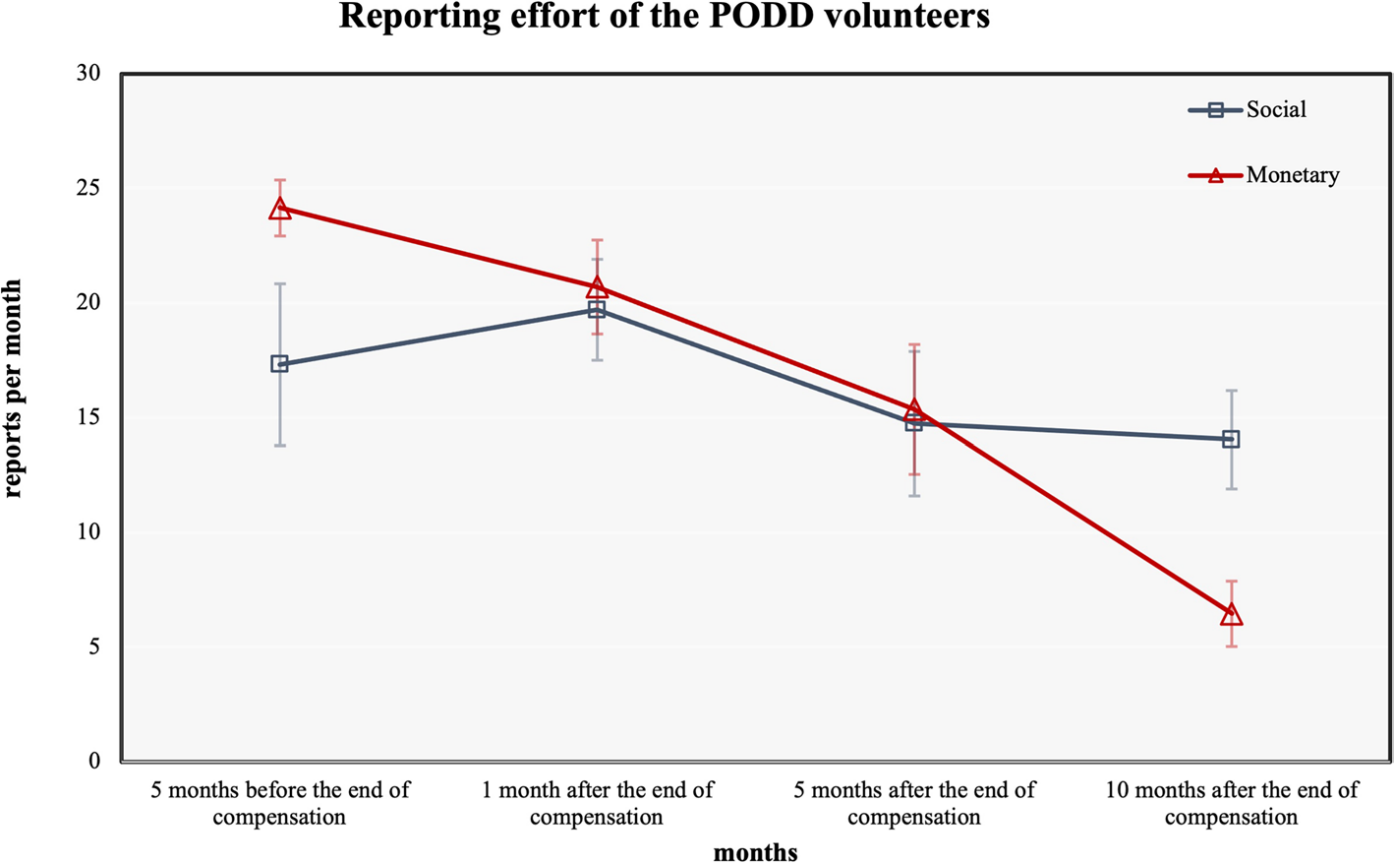
The reporting effort of PODD Volunteers comparing between monetary market group and social market group. In the long-term period, a social market group showed significantly higher number of report than monetary market group

As shown in Fig. 2, question 1 was answered using a t test to compare the reports of the two groups in Period 1. The results indicate that the average reports of the monetary market group were 24.15 reports per month, while those of the social market group were 17.32 reports per month (Table 2). During the time in which the volunteers in the monetary market group were paid, the monetary market group's effort was significantly higher than that of the social market group, the *p*-value, *p* = 0.03 (Table 2). Therefore, the first assumption was confirmed by these results**.**

**Table 2:** A t test results comparing social market group and monetary market group in the period 1 to 4

	Social group *n* = 5		Monetary group *n* = 12		*t*- test		
	Mean	SD	Mean	SD	t	df	*p*-value
Period 1	17.32	7.88	24.15	4.23	-2.36	15	0.03*
Period 2	19.70	4.94	20.70	7.09	-0.29	15	0.78
Period 3	14.75	7.03	15.36	9.81	-0.13	15	0.90
Period 4	14.05	4.78	6.46	4.92	2.92	15	0.01*

^*^(*p* < 0.05)

Then, question 2, it was answered using repeated-measurement ANOVA. The results indicate that the reports of the monetary market group showed a statistically significant decreasing trend, *F*_3,13_ = 25.51, *p* < 0.001 (Table 3). The descriptive statistics in Table 2 indicate the decreasing trend of the monetary market group’s reports. Moreover, the results indicate that there were no significant differences in reporting effort in the social market group throughout the experimental period, *F*_3,13_ = 2.00, the *p*-value, *p* = 0.16 (Table 3). The second assumption was therefore confirmed by these results**.** The increased reports within the social market group in Period 2 (one month after the end of compensation) is not statistically significant, and the result is presented in Table 3. The statistics in Table 3 demonstrate that there is no significant difference in the number of reports within the social market group across the four periods of time. However, within the monetary market group, there is a statistically significant difference in the number of reports.

**Table 3:** The repeated measurement ANOVA results social market group and monetary market group

Group		Value	F	*p*-value
Social Group	Pillai's trace	0.32	2.00	0.16
	Wilks' lambda	0.68	2.00	0.16
	Hotelling's trace	0.46	2.00	0.16
	Roy's largest root	0.46	2.00	0.16
Market Group	Pillai's trace	0.86	25.51	< 0.001**
	Wilks' lambda	0.15	25.51	< 0.001**
	Hotelling's trace	5.89	25.51	< 0.001**
	Roy's largest root	5.89	25.51	< 0.001**

^**^(*p* < 0.001)

Finally, question 3 was answered using a t test to compare the reporting of the two groups in Period 4. The results indicate that after the compensation was terminated for 10 months, the monetary market group had a significantly lower reporting effort than the social market group, the *p*-value, *p* = 0.01(Table 2). The average of the reporting effort of the monetary market group was 6.458 times per month, while that of the social market group was 14.05 times per month (Table 2). Thus, the third assumption was also confirmed by these results**.**

## Discussion

According to the findings, this study concentrated on the difference between monetary and social (nonmonetary) incentives for animal health reporting. This study utilized data from the PODD project. According to the preliminary findings, monetary incentives lowered the effort of volunteers in the monetary market group after the payment period ended and in the long term. Social incentives, on the other hand, could be able to keep the volunteers in the social market group working consistently throughout the study.

When the payment is still being made available, monetary incentives are an effective method of enhancing effort. The findings of this study show that throughout the payment period, the monetary market group reported more than the social market group (*p* < 0.05). The volunteers' level of reporting effort in the monetary market was relevant to the payment they earned. However, after the payment was withdrawn, the reporting effort of the monetary market group decreased (*p* < 0.001), whereas the social market group reported more consistent efforts over time (*p* = 0.16). The effort of the monetary market group has continuously declined over all time periods. The monetary market group's effort had decreased by 73% ten months after payment ended (*p* < 0.001), and this was a 54% decrease from the social market group's result. (*p* < 0.05). This indicates that once the volunteers were compelled to enter the monetary market, they were unable to revert to the social market, even if the duration was 10 months, whereas the social incentive was able to drive the social market group to produce reports at all times without payment. We also assume that the negative effect of monetary incentives remained and that the number of reports per month would decrease after the payment period. However, 10 months after the end of compensation, the PODD project decided to change the reporting policy. Volunteers no longer had to report on a daily basis. This study decided to compare this period of time as the end of the experiment because subsequently, the daily report requirement was canceled. Monetary incentives not only reduced the effort of the monetary market group in the long term, but also became an obstacle to its return to a subsequent social market relationship. As the long-term negative impact of the monetary market, even if we want the volunteers to return to the social market, it is not easy for them to do so.

Under monetary markets, people lose their motivation when the level of compensation is not sufficient to meet their expectations. Under Fiske's 1992 theory, changes in social relationships, whether from CS, AR, or EM, into an MP relationship, are equivalent [14]. Under monetary market conditions, compensation can be compared to the effect of the wind on a sailing boat – the boat only sails only as long as the wind is blowing [16]. The results demonstrate the difference volunteer behaviors in the monetary market group compared to the standard labor model of economics. At the end of the compensation period, it was notable that the efforts of the monetary market group did not vanish, which is the opposite of the prediction of the standard labor model of economics. The atmosphere of social activity may have reduced the decline in efforts for a while but eventually, it was reduced by the impact of the monetary market. Moreover, after 10 months of compensation, the social market groups had a higher report rate than the monetary market groups. The result does not align with the standard labor model of economics [10]. The results of this study show a different dimension for understanding human motivation that is different from the rationality of economic assumptions. On the other hand, the results confirm that monetary incentives are more influential than social incentives. Social incentives cannot be used for a long time after monetary incentives are used [10, 15, 16].

In addition, our findings indicate that monetary markets can be used to motivate people in the short period. As long as there is still a payoff, using monetary incentives has a significant influence on increasing effort. Simultaneously, it forces people to make decisions about the costs and benefits of their efforts, encouraging them to focus on self-interest instead of social welfare, whereas a social market group's volunteers perform for their community without expectation of reciprocation. In conclusion, the study found that volunteers were more motivated to work unpaid over time than when they were paid initially and then stopped. In terms of sustainability, social incentives have been found to be more effective than monetary incentives. Social incentives can not only drive long-term activities, but they can also be kept at a cheap price. The social markets, on the other hand, are extremely vulnerable to monetary market interference. Similarly, volunteers' motivation is relatively consistent throughout time. In the participatory disease surveillance system, it is motivated by the use of social markets instead of monetary markets. This enables developers of participatory disease surveillance systems to gain a better understanding of the overall picture, including the role of incentives for participants and stakeholders.

The limitations of this study were the limitation of the internet signal and the variation in livestock which constrained this study design in the central part of Chiang Mai Province. Moreover, this study was a long-term study of an actual project that involved studying a real situation. It involved handling a large budget and the PODD project had to run the process on time. This study could not compare the number of reports before compensation was started because of the motivation policy of the PODD project. The small sample size available in the PODD project was one of the study's limitations, and it also limited the transferability of the results. There are some challenges for future studies, such as differences in culture or geography as the tools for comparing the impact of social and monetary markets. In this study, we focus only on the number of reports. Further studies could be extended to the quality of reports to gain a greater understanding of the effect of monetary and social incentives on participatory disease surveillance systems.

## Conclusions

By focusing on human behavior as a key success component for the systems, in terms of motivation, monetary incentives can enhance reporting effort, but they come at a significant cost and have a detrimental long-term impact. When the payment is withdrawn, the effort will gradually decrease. Even over the long run, this study demonstrates that there is no likelihood of returning to social incentives after monetary incentives have been applied. This study highlights a behavioral knowledge gap. The economically rational assumption cannot be utilized to explain individual behavior in the PODD project on its own. It demonstrates a lack of awareness of human cognitive bias in predicting human decisions. Understanding human behavior has the major benefit of improving the planning and construction of effective digital animal disease surveillance systems.

According to the study's discussions and conclusions, it is critical for policymakers, government agencies, and surveillance system developers to have a holistic understanding of the surveillance process, which encompasses many parties. Authorities must be aware of the two types of motivation: social and monetary incentives and select the motivation that is most suitable for their project or system. In the long term, social incentives, particularly for low-budget projects, should be an effective approach to motivate reporters. Because social rewards have a lower cost than monetary incentives, Authorities must be aware of the social incentive vulnerability if social incentives are utilized to motivate participants' effort in a future project. However, if monetary incentives are used, authorities must be aware that monetary incentives necessitate a significant budget in order to improve the efforts of reporters as well as the opportunity cost of losing the option to use social incentives in the future.

## Data Availability

All data generated and analyzed during the current study are available from PODD center, Chiang Mai University on reasonable request. If anyone who want to request the data, poddcenter.cmu@gmail.com can be contacted.

## Abbreviations

PODD: Participatory One Health Disease Detection project
CS: Communal sharing
AR: Authority ranking
EM: Equality matching
MP: Market pricing
ID: Identification
